# Newly graduated nurses’ empowerment regarding professional competence and other work-related factors

**DOI:** 10.1186/s12912-016-0143-9

**Published:** 2016-03-24

**Authors:** Liisa Kuokkanen, Helena Leino-Kilpi, Olivia Numminen, Hannu Isoaho, Mervi Flinkman, Riitta Meretoja

**Affiliations:** Helsinki University Hospital, Finland, Corporate Headquarters, Henkivartijantie 18, FI-000620 Helsinki, Finland; Department of Nursing Science, Turku University Hospital, University of Turku, Turku, Finland; Station Ltd, Salo, Finland; Helsinki University Hospital, Finland, Corporate Headquarters, Turku, Finland; University of Turku, Department of Nursing Science, Turku, Finland

**Keywords:** Nurse empowerment, Professional competence, Work-related factors, Quantitative study, Workforce issues, Newly graduated nurses

## Abstract

**Background:**

Although both nurse empowerment and competence are fundamental concepts of describing newly graduated nurses’ professional development and job satisfaction, only few studies exist on the relationship between these concepts. Therefore, the purpose of this study was to determine how newly graduated nurses assess their empowerment and to clarify professional competence compared to other work-related factors.

**Methods:**

A descriptive, cross-sectional and correlational design was applied. The sample comprised newly graduated nurses (*n* = 318) in Finland. Empowerment was measured using the 19-item Qualities of an Empowered Nurse scale and the Nurse Competence Scale measured nurses’ self-assessed generic competence. In addition to demographic data, the background data included employment sector (public/private), job satisfaction, intent to change/leave job, work schedule (shifts/business hours) and assessments of the quality of care in the workplace. The data were analysed statistically by using Spearman’s correlation coefficient as well as the One-Way and Multivariate Analysis of Variance. Cronbach’s alpha coefficient was used to estimate the internal consistency.

**Results:**

Newly graduated nurses perceived their level of empowerment and competence fairly high. The association between nurse empowerment and professional competence was statistically significant. Other variables correlating positively to empowerment included employment sector, age, job satisfaction, intent to change job, work schedule, and satisfaction with the quality of care in the work unit. The study indicates competence had the strongest effect on newly graduated nurses’ empowerment.

**Conclusions:**

New graduates need support and career opportunities. In the future, nurses’ further education and nurse managers’ resources for supporting and empowering nurses should respond to the newly graduated nurses’ requisites for attractive and meaningful work.

## Background

The nursing practice environment is often very complex with demands on high-level competence and capability to collaborate as a member of the care team. For the newly graduated nurse (hereafter NGN), this environment may be daunting [1, 2]. NGNs’ first years of practice signify going through an important phase of building confidence and professional identity. Earlier studies show that many nurses change their job or even leave the profession within the first years of practice [3, 4]. There are several factors influencing the NGNs’ turnover intentions. When NGNs are satisfied with their jobs and feel empowered, the odds of turnover intent decrease [5]. Findings on nurses’ perceptions of their work environment also show that NGNs receive more support from their nurse managers than the other nurses. However, NGNs describe disempowering experiences, such as feeling undervalued by other nurses, feeling overwhelmed and having their opportunities blocked [6].

Apart from support, NGNs want feedback on their competence and their management of different care situations, which has a profound effect on nurses’ work motivation and job satisfaction [7]. Generational differences in nurses’ empowerment may affect their perceptions of their work environment. Further, the impact of empowerment on affective commitment for Generation X and Baby Boomers compared with Generation Y nurses differs [8, 9]. In a study exploring employers’ perceptions of the level of knowledge, competence and professionalism of NGNs, the employers identified what could be attributed to a ‘new generation of nurses’ [7, 10]. Employers valued NGNs’ assertiveness and their ability to solve problems. A major strength of the NGNs was their willingness to continue learning, their willingness to seek out information and to assume responsibility in compensation for their lack of knowledge. Work-related empowerment is an important element among NGNs trying to function in educational and ever-changing health care settings. Nurse education programmes enhancing empowerment may affect psychological empowerment, competence and job productivity [11].

Besides clinical skills, NGNs need a strong sense of professional values and identity. The responsibility falls on educational institutions and nursing organizations to empower NGNs and to enter them into the profession [9, 12]. Although both nurse empowerment and competence are fundamental concepts of NGNs’ professional development and job satisfaction, only few studies exist on the relationship between these concepts.

### Theoretical framework

Empowerment is an abstract and dynamic concept, which is not feasible to confine exactly in space or time. It has been described both as a state and a process. The core of the empowerment process is to strive persistently for the greatest attainable mastery over one’s living conditions and over the relevant arrangements [13, 14]. Lacking coherent and unambiguous definitions, the concept has been provided with characteristics such as individual efficiency, competence and coping abilities. Collectively empowerment can be traced to mutual support. Its negation has been depicted as powerlessness, hopelessness, disablement or frustration and, at its worst, subjugation and oppression [15, 16].

Empowerment has been described from three aspects using critical social, organizational and psychological theories [16, 17]. On the one hand, empowerment has been portrayed as an essential part of human nature and development, and on the other hand it has been described as an aspect to organizational effectiveness and quality. The psychological empowerment based on personal development examines empowerment from the viewpoint of individuals [16]. Here empowerment is a prolonged development and learning process, it is dynamic and synergetic, involving both positive and negative bearings. Personal reflection is a precondition for accumulation of knowledge and individual growth [18, 19]. To understand the meaning of empowerment, we need to look at the concept of power. Power and empowerment are interwoven in multiple, often subconscious or even inexplicable ways. Power as a motivational sense refers to an intrinsic need for self-determination and to a belief in personal self-efficacy. In a legal sense it means authority [20]. As a counterpoise to power, a person is both the subject and the object because power relationships are always reciprocal. For instance, even though power in organizations has traditionally been regarded as a means of control, it could, from the individual’s viewpoint, mean managing to get along with given situations: individuals feel they possess power. An empowered individual does not report having attained more power but having become empowered [16, 21]. Powerlessness is viewed as a construction of continuous interaction between the person and the person’s environment [13].

From the viewpoint of an individual nurse, the empowerment process consists of both critical introspection and outside guidance leading to an appropriate modifying action. Experiences of mastery prove effective as these performance successes strengthen self-beliefs and capability [22]. Capable people are able to show competencies in novel and complex situations. NGNs are presumably persons guided by their social abilities and willingness to act in accordance with their human values. The empowered nurse possesses yet something more, something that renders self-esteem, successful job performance, and possible progress. According to Davis and Hase [23], competency measures previous performance, whereas capability and empowerment focus on the future, on circumstances still unknown.

Studies have demonstrated that empowered nurses have a more positive attitude towards their work, are more satisfied with their job, and experience moral distress less often than others [24–26]. Furthermore, organizational justice and empowerment have a positive correlation [27]. Empowerment is also related to career development. Nurses wish to be involved and exert influence within their work at a personal level. Studies confirm that nurses who are able to apply their skills at work and develop their work assess their level of empowerment higher than other nurses [28]. A study of outcomes of a formal leadership development programme provides evidence that theoretical empowerment frameworks and strategies can empower nurse leaders, potentially resulting in staff empowerment [29]. The content of concepts describing empowerment and competence appear nearly identical. However, competence is based on skills, attitudes and values into specific health care contexts, whereas psychological empowerment refers to personal development, self-efficacy and self-belief.

Thomas and Velthouse [20] developed the psychological model of empowerment from the perspective of motivation theory. Thomas and Velthouse’s psychological model formed the framework for the study introducing theme-based interviews relevant to empowerment [30]. In this Kuokkanen’ et al. qualitative study, five categories describing the qualities of an empowered nurse (QEN) emerged: moral principles, personal integrity, expertise, future-orientedness, and sociability (Table 1, [30]). These five categories were adopted as the conceptual framework for the questionnaire used in this study.

**Table 1:** Qualities of an empowered nurse (QEN) (*n* = 318, range 1–5; 1 = least, 5 = most)

Mean	Sd	Cronbach’s Alpha	
Moral principles	*4.54*	*0.49*	*0.75*
Respect for individuals	4.76	0.55	
Honesty	4.59	0.58	
Equity	4.23	0.66	
Personal integrity	*3.90*	*0.60*	*0.74*
Mentally resourceful	3.94	0.76	
Courageous, assertive	3.82	0.82	
Able to act under pressure	3.61	0.87	
Broadminded, flexible	4.22	0.71	
Expertise	*3.70*	*0.58*	*0.73*
Autonomous	3.66	0.84	
Has personal power	3.20	0.97	
Skilful	3.96	0.81	
Knowledge	3.85	0.75	
Personally responsible	3.87	0.80	
Future-orientedness	*3.69*	*0.64*	*0.67*
Innovative, creative	4.08	0.86	
Enthusiastic promoter	3.38	0.92	
Forward thinking	3.78	0.95	
Sociability	*3.94*	*0.65*	*0.74*
Open-minded	4.05	0.86	
Respected by others	3.88	0.79	
Socially responsible	3.93	0.74	
Total	*3.95*	*0.46*	*0.72*

## Methods

### Aim

This study explored NGNs’ empowerment and its associations with their self assessed professional competence, turnover intentions, work schedule, satisfaction with the quality of care in the unit, and demographic factors.

### Research design and instruments

A descriptive, cross-sectional and correlational design was applied. Empowerment was measured using the 19-item Qualities of an Empowered Nurse (QEN) scale [30]. The instrument was based on the five categories representing moral principles, personal integrity, expertise, future-orientedness, and sociability obtained from an earlier qualitative study ([30], Table 1). ‘Moral principles’ reflects human values in nursing (‘Respecting other people is the most important principle that guides my work’, ‘I am fair and just’, 30). ‘Personal integrity’ refers to mastery of one’s own life and is also expressed by recognizing one’s own resources (‘I am brave’, ‘I can tolerate uncertainty and stress’). ‘Expertise’ manifests itself as professional competence and as a wide range of knowledge (‘I am skilled in my work’, ‘I am willing to bear a lot of personal responsibility’). ‘Future-orientedness’ involves creativity and innovation (‘I have many creative ideas which could be used in my work’, ‘I continually set myself new challenges/targets’). ‘Sociability’ refers to socially skilled nurses who are flexible and have the ability to create a positive ambience in the workplace (‘I find it easy to form new relationships’, ‘My work colleagues value me’). The content, criterion-related and construct validity of the instrument has been tested in earlier studies [27, 31, 32]. Cronbach’s alpha coefficients have varied between 0.87–0.88 [24, 27, 31]. For this sample, Cronbach’s alpha coefficient for the QEN subcategories ranged from 0.67 to 0.75. The items were rated on a 5-point Likert scale, ranging from 1 (totally disagree) to 5 (totally agree) with high values of the average of subcategory scores indicating high empowerment. In addition to demographic data, the background data included employment sector (public/private), job satisfaction, intent to change/leave job, work schedule (shifts/business hours) and assessments of the quality of care in the workplace.

Competence was used here as an independent dichotomous variable to divide NGNs into higher and lower competence level groups using the median score (VAS = 65). Nurse competence was measured with the Nurse Competence Scale [33]. It is a generic competence instrument based on Benner’s [34] From Novice to Expert theoretical framework according which experience is a prerequisite for becoming an expert nurse. The NCS comprises seven theoretical categories: helping role (7 items), teaching–coaching (16 items), diagnostic functions (7 items), managing situations (8 items), therapeutic interventions (10 items), ensuring quality (6 items) and work role (19 items). The level of competence is measured with a Visual Analogy Scale (VAS, 0 = very low −100 = very high) [33]. The content, criterion-related and construct validity of the instrument has been tested in earlier studies [33]. Cronbach’s alpha coefficients have varied between 0.72 and 0.93 [33, 35] indicating strong internal consistency. In this study, Cronbach's alpha coefficient for the NCS subcategories ranged from 0.76 to 0.92.

Work-related factors concerning turnover intentions (2 questions) and job satisfaction (4 questions) were also analysed as independent variables. The demographic data included previous professional education, age and gender, employment sector, work schedule, and quality of care in NGNs’ work units.

### Sample and data collection

The target group comprised NGNs registered between November 2011 and October 2012 in Finland. This study is part of the ‘Newly Educated Workers’ (NEW) research project. The member registers of the Finnish Nurses’ Association and the Union of Health and Social Care Professionals were used to retrieve the eligible participants including their e-mail addresses. The length of their work experience as registered nurses could not exceed 12 months. An e-mail consisting of electronic questionnaire including QEN and NCS instruments, questions concerning participants’ demographics, work-related background variables, and a cover letter was sent to 1050 nurses in the beginning of November 2012. The cover letter included information about the purpose of the study, reporting of the findings, and issues related to research ethics. In addition, we sent two reminder letters to the participants.

### Statistical analysis

The data analysis was performed using the Statistical software NCSS version [36]. The sum variables were formed according to the theoretical categories (Table 2). The reliability of sum variables was measured by calculating Cronbach’s alpha coefficients and examining the compatibility of single questions with the instruments by using an item analysis. For multi-group background variables, comparisons of groups were made by using the Spearman correlation test and the One-Way (ANOVA) and Multivariate Analysis of Variance (MANOVA). The observed significance levels <0.05 were considered statistically significant, and power values ≥ .800 (at alpha = .05) were regarded as strong.

**Table 2:** Spearman correlations between qualities of an empowered nurse and nurse competence (*n* = 318, range 1–5; 1 = least, 5 = most)

Empowerment	Competence (NCS)	
Categories (QEN)	Correlation	Significance
Moral principles	0.214	<0.001
Personal integrity	0.358	<0.001
Expertise	0.481	<0.001
Future-orientedness	0.449	<0.001
Sociability	0.345	<0.001
All categories	0.482	<0.001

### Ethical considerations

The ethical approval was obtained from the Ethics Committee of the University of Turku (reg. 15/2012). The Finnish Nurses’ Association and the Union of Health and Social Care Professionals granted their permission to conduct the study. Nurses were provided with a full explanation of the purpose of the study and were informed about researchers’ commitment to research ethics. Returning a completed questionnaire was interpreted as consent to participate in the study [37].

## Results

### Sample

In total 318 (30 %) NGNs returned the questionnaire. The respondents were mostly female (91 %) with an average of 9 months since their graduation and 8 months of work experience as registered nurses. Half of the NGNs (51 %) had previous professional education, mainly from the health care sector (64 %). Most NGNs were employed by the public sector (88 %) and over half of them did three-shift work (58 %). Many NGNs had never or seldom thought of changing their work place (66 %). They were satisfied with their current job (84 %), nursing profession (86 %) and the quality of care in their work unit (90 %).

### Empowerment categories

The NGNs’ perceptions of their empowerment ranked relatively high. The means for the five empowerment subcategories (QEN) varied from 3.70 to 4.54 (mean 3.95, SD 0.46). Nurses assessed ‘moral principles’ as the highest category and ‘future-orientedness’ as the lowest (Table 1).

### Empowerment and background variables

The relation of background variables to empowerment (QEN) was tested using the Spearman correlation test and the One-Way Analysis of Variance (ANOVA). The correlation between competence and the five QEN subcategories was statistically significant (Spearman 0.482, *p* < 0.001, Table 2).

Higher self-assessment of professional competence (*p* < 0.001), older age (over 25 years, *p* = 0.007), and having no plans to change jobs (*p* < 0.001) correlated positively with the NGNs’ level of empowerment. Of these variables, competence (NCS) had the strongest effect on empowerment (Table 3). Working status (permanent/temporary) or previous health care education did not have a statistically significant relation to the level of empowerment.

**Table 3:** Significant main effects related to NGNs’ assessments of their empowerment (QEN) and background variables (*n* = 318, ANOVA)

Variables	Response format	Adjusted mean of QEN	Sum of squares	F-Ratio	*p*-value
(Alpha = 0.05)					
Professional competence	VAS > 65	4.08	8.86	52.58	*p* < 0.001
	VAS ≤ 65	3.73			
Planning to change job	Never/seldom	4.00	2.60	15.43	*p* < 0.001
	Often	3.80			
Age	25 years and over	3.97	1.26	7.51	*p* = 0.007
	Under 25 years	3.84			

According to the multivariate tests (MANOVA), variables related to empowerment (QEN) categories included competence, employment sector, age, job satisfaction, intent leave job, work schedule, and quality of care. Those NGNs with higher competence (*p* < 0.001) and working in the public sector (*p* = 0.001) rated the ‘moral principles’ category higher than the others. Older respondents (over 25 years, *p* < 0.001), NGNs with no plans to change their job (*p* = 0.018), and those not satisfied with the quality of care in their work unit (*p* = 0.047) rated ‘personal integrity’ higher than the others. Furthermore, NGNs with more ‘personal integrity’ assessed their competence higher (*p* < 0.001) than the others. Those with a high degree of satisfaction with nursing profession (*p* = 0.004), those working on business hours (*p* = 0.003) and nurses with high competence rated ‘expertise’ higher than the others. ‘Future-orientedness’ rated higher among NGNs satisfied with nursing profession (*p* = 0.039), NGNs with no intent of changing work place (*p* = 0.005) and those with high competence. Moreover, NGNs satisfied with their job (*p* = 0.011), those with no plans to change their job (0.004), and nurses with high competence (*p* < 0.001) rated ‘sociability’ higher than the others (Table 4).

**Table 4:** Significant effects on nurses’ assessments of their empowerment categories (moral principles, personal integrity, expertise, future-orientedness, sociability) to background variables (*n* = 318, MANOVA)

	Moral principles					
Variables	Response format	Adjusted mean	Sum of squares	F-Ratio	*p*-value	Power (Alpha = 0.05)
Competence	VAS > 65	4.62	3.82	17.31	*p* < 0.001	0.99
Employment sector	Public	4.55	1.48	6.72	*p* = 0.01	0.73
	Personal integrity					
Variables	Response format	Adjusted mean	Sum of squares	F-Ratio	*p*-value	Power (Alpha = 0.05)
Competence	VAS > 65	4.01	4.43	15.98	*p* < 0.001	0.97
Age	Over 25 year	4.01	4.6 4	16.76	*p* < 0.001	0.98
Planning to change job	Never/seldom	3.94	1.57	5.66	*p* = 0.018	0.66
Satisfied with the quality in work unit	Not agree	3.93	1.10	3.98	*p* = 0.047	0.51
	Expertise					
Variables	Response format	Adjusted mean	Sum of squares	F-Ratio	*p*-value	Power (Alpha = 0.05)
Competence	VAS > 65	4.64	12.35	46.3	*p* < 0.001	1.0
Work schedule	Business hours	3.80	3.19	5.99	*p* = 0.003	0.88
Satisfied with nursing profession	Yes	3.75	2.22	8.34	*p* = 0.004	0.82
	Future-orientedness					
Variables	Response format	Adjusted mean	Sum of squares	F-Ratio	*p*-value	Power (Alpha = 0.05)
Competence	VAS > 65	3.78	13.81	40.8	*p* < 0.001	1.0
Planning to change job	Never/seldom	3.67	2.76	8.17	*p* = 0.005	0.81
Satisfied with nursing profession	Yes	3.67	1.45	4.29	*p* = 0.039	0.54
	Sociability					
Variables	Response format	Adjusted mean	Sum of squares	F-Ratio	*p*-value	Power (Alpha = 0.05)
Competence	VAS > 65	3.95	5.96	15.8	*p* < 0.001	0.98
Planning to change job	Never/seldom	3.93	3.24	8.61	*p* = 0.004	0.83
Satisfied with current job	Yes	3.94	2.49	6.62	*p* = 0.011	0.73

## Discussion

### Findings

Based on many earlier studies on empowerment, nurses are moderately empowered [5, 38]. In this study, however, newly graduated nurses assessed their empowerment fairly high. This confirms the earlier study findings on young nurses considering themselves more empowered than the older [31]. Likewise, Ning et al. [39] discovered older nurses were less empowered than the younger. Presumably NGNs’ short experience of nursing practice, different learning environments and different clinical competence expectations has an impact on their assessments of their empowerment [40, 41]. For example, young nurses’ assertiveness and their willingness to seek out information as well as assuming responsibility for their lack of knowledge [22] could influence their reflections on their empowerment. A recent study [40] shows graduate nurses could assess their overall ability and skills remarkably well. According to this study, graduates were even more critical of their performance than their employers, but tended to focus on specific skills. Our study indicates competence had the strongest effect on NGNs’ empowerment. Earlier studies confirm that nurses who are able to apply their skills at work assess their level of empowerment higher than other nurses [27, 28].

As to the highest and lowest empowerment subcategories, this study aligns with earlier studies [27, 31, 32]. NGNs had the highest rating on ‘moral principles’ and the lowest on ‘future-orientedness’. The NGNs’ high rating on ‘moral principles’ may well be the result of good education of ethical principles in nursing care [42]. According to earlier research, nurses with higher perceived empowerment experience moral distress less often than others [25]. The low rating on ‘future-orientedness’ could lead to a diminished role of innovativeness and problem solving. Education and management should encourage innovative behaviour, such as generation and realization of ideas and mobilization of support. This could also be included in the competencies of nursing curricula. An earlier study [43] suggests a strong relationship between psychological empowerment and innovative behaviour.

This study found a significant positive correlation between the five nurse empowerment (QEN) subcategories and professional competence. This presents a new finding although the relationship is very logical. Interestingly, NGNs rating high scores for ‘personal integrity’ assessed high level of competence as well. People’s beliefs about their efficacy are fundamental determinants of human motivation, effect and action [19, 44]. The acceleration of confidence and capability development in NGNs assures their accelerated competence acquisition is applied. The results suggest that those NGNs rating their ‘personal integrity’ high were not satisfied with the quality of care in their work units. This is a new finding requiring further research.

The results on this study indicate NGNs’ satisfaction with their current job and the nursing profession. The results also show a significant relationship between empowerment and job satisfaction paralleling several earlier studies [7, 38, 45]. The underlying reasons for job satisfaction or discontent are manifold. According to previous research, job satisfaction is related to both work environmental factors and personal characteristics. A recent paper [46] suggests that on the one hand NGNs reported consistent dissatisfaction with their work environment and the hospital system in general as well as declining satisfaction with staff scheduling. On the other hand, they were satisfied with the opportunity of participating in further education and developing themselves as health care professionals. The positive correlation between empowerment and job satisfaction is an important finding. Psychological empowerment has a mediating effect on a number of organizational and outcome variables, such as anticipated turnover and organizational commitment [5, 17]. Feeling empowered means not only that individuals find their work meaningful, but they also find it to be a source of intrinsic satisfaction because they feel competent and autonomous, and because they believe they have a positive impact on the organization [44, 45].

In accordance with many previous studies [46], the results of this study confirm that NGNs considering themselves more empowered had no plans to change their job. The risk of NGNs leaving their employment is very high within the first year of graduation [3, 47]. Nurses are willing to seek a new job or even an entirely new profession. The number of young nurses who had considered leaving the profession or changing their job has been markedly high in earlier research [4, 5, 47, 48]. A shortage of nurses is a global phenomenon, as also indicated by the International Council of Nurses [49]. The magnitude of factors contributing to the shortage results in the need to resolve the problem from multiple perspectives. Nurse managers hold an important position of enhancing nurse empowerment [50]. New graduates need support and career opportunities. The same applies to having influence and decision-making authority over their work and its environment. In particular, young nurses benefit from the support of their work environment [51]. Moreover, generational differences in nurses’ empowerment may also affect their perceptions [9, 10]. Further research is needed to find the possible direct link between nurse empowerment and nurse retention.

### Study limitations and strengths

The instruments used had been previously tested and their psychometric properties established making them applicable to this study [37]. The response bias is a cause of concern in the use of self-reported questionnaires. According to Paulhus [52], the basic elements of self-report include impression management and self-deception. This phenomenon is complicated and extremely hard to detect and control. There is usually no need to control socially desirable responding when answering is anonymous, and the respondents remain unidentifiable [52]. The anonymity of respondents and their workplace ensured confidentiality thus establishing the validity and reliability of the study.

The low response rate renders the interpretation of the findings somewhat problematic. Low response rates are often associated with posted and even more with e-mailed surveys. The target group itself may also display a data bias because presumably not all NGNs have registered as members of the Finnish Nurses’ Association or the Union of Health and Social Care Professionals. However, after previous studies, the respondents provided a demographically and geographically representative sample of NGNs allowing proper statistical analyses.

Furthermore, self-reported data impose another limitation on the study. After 9 months of graduation, NGNs’ work environments and experiences of nursing care might have varied greatly. Evidently, self-assessments only consider one aspect of this phenomenon and further research is needed to assess these factors from the viewpoint of nurse leaders and NGNs’ colleagues. Consequently, the question on nurses’ perceptions of their empowerment still requires further research. While empowerment and competence are fundamental concepts in developing nursing care, further research is needed to find the connections and differences of these concepts.

## Conclusions

Nurse managers should respond to the challenge of empowering NGNs and provide means of supporting them to find their work more meaningful and attractive. Both nurse education and management should encourage nurses in innovativeness and problem solving, and these could also be included in the competencies of nursing curricula. The responsibility for providing graduate nurses with a sense of empowerment during their first years of practice, establishing a strong professional identity and making their adaptation to their profession successful rests with education, nurse managers and colleagues.

## Abbreviations

NCS: the nurse competence scale
NGN: newly graduated nurses
QEN: qualities of an empowered nurse
